# *In Vivo* Evidence for Platelet-Induced Physiological Angiogenesis by a COX Driven Mechanism

**DOI:** 10.1371/journal.pone.0107503

**Published:** 2014-09-19

**Authors:** Ian M. Packham, Steve P. Watson, Roy Bicknell, Stuart Egginton

**Affiliations:** 1 Centre for Cardiovascular Sciences, School of Clinical and Experimental Medicine, College of Medical and Dental Sciences, University of Birmingham, Edgbaston, Birmingham, United Kingdom; 2 Centre for Cardiovascular Sciences, School of Immunity and Infection, College of Medical and Dental Sciences, University of Birmingham, Edgbaston, Birmingham, United Kingdom; 3 School of Biomedical Sciences, Faculty of Biological Sciences, University of Leeds, Leeds, United Kingdom; University of Illinois at Chicago, United States of America

## Abstract

We sought to determine a role for platelets in *in vivo* angiogenesis, quantified by changes in the capillary to fibre ratio (C∶F) of mouse skeletal muscle, utilising two distinct forms of capillary growth to identify differential effects. Capillary sprouting was induced by muscle overload, and longitudinal splitting by chronic hyperaemia. Platelet depletion was achieved by anti-GPIbα antibody treatment. Sprouting induced a significant increase in C∶F (1.42±0.02 *vs.* contralateral 1.29±0.02, *P*<0.001) that was abolished by platelet depletion, while the significant C∶F increase caused by splitting (1.40±0.03 *vs.* control 1.28±0.03, *P*<0.01) was unaffected. Granulocyte/monocyte depletion showed this response was not immune-regulated. VEGF overexpression failed to rescue angiogenesis following platelet depletion, suggesting the mechanism is not simply reliant on growth factor release. Sprouting occurred normally following antibody-induced GPVI shedding, suggesting platelet activation *via* collagen is not involved. BrdU pulse-labelling showed no change in the proliferative potential of cells associated with capillaries after platelet depletion. Inhibition of platelet activation by acetylsalicylic acid abolished sprouting, but not splitting angiogenesis, paralleling the response to platelet depletion. We conclude that platelets differentially regulate mechanisms of angiogenesis *in vivo*, likely *via* COX signalling. Since endothelial proliferation is not impaired, we propose a link between COX1 and induction of endothelial migration.

## Introduction

**Figure 1:**
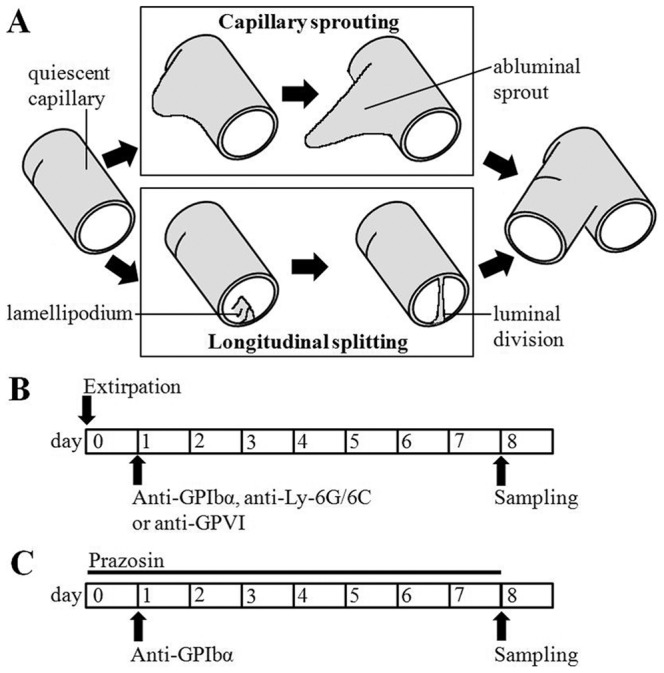
Different forms of angiogenesis and experimental protocols. A) Mechanisms of capillary sprouting and longitudinal splitting. B) Extirpation induced endothelial sprouting, with anti-GPIbα, anti-Ly6G/6C or anti-GPVI administration 24 h after surgery. C) Prazosin induced longitudinal splitting with anti-GPIbα. Sampling always occurred on the eighth day.

At least two mechanisms of new vessel formation (angiogenesis) are now recognised, namely capillary sprouting and longitudinal splitting [1] (Figure 1A). Sprouting angiogenesis involves abluminal outgrowth, when mechanical deformation of capillaries stimulates endothelial cell (EC) activation and proliferation. Formation of filopodia and breakdown of the basement membrane results in expansion of the capillary bed under reduced fluid shear stress (FSS) [2]. Sprouting can be induced experimentally by extirpation of a skeletal muscle, leading to overload of the remaining synergists. In contrast, longitudinal splitting arises from sustained increases in blood flow, which stimulates formation of luminal lamellipodia that fuse, eventually dividing capillaries. Experimentally, splitting angiogenesis can be induced by administration of α_1_-adrenoceptor antagonists, such as prazosin [3], causing vasodilatation to chronically elevate capillary FSS. Both forms of angiogenesis may be seen in various pathologies, apparently determined by the local shear environment [4], but possibly also by recruitment of endothelial mitosis that is markedly lower in neocapillary formation by splitting. There is no substantive difference between mouse and rat in the angiogenesis models utilised [4]. Although platelets are implicated as mediators of pathological angiogenesis, such as that driven by inflammation or tumour vascularisation [5], their contribution to non-reparative, physiological angiogenesis during tissue remodelling has not been studied.

Platelet involvement is thought to be due to: (i) release of growth factors or (ii) platelet-vessel interactions [5]. Platelets contain α-granules, dense granules, and lysosomes. The α-granule content includes both pro- and anti-angiogenic growth factors (including VEGF, FGF-2, PDGF, PF4, TSP-1 and endostatin) that are important regulators of some tumour growth [6], [7]. Platelet releasate has been linked with *in* vitro and *in* vivo angiogenesis, involving neuropeptide Y and the angiopoietin pathway [8]–[10]. The mechanism of growth factor sequestration is unclear [11], but may involve endocytosis of circulating vascular endothelial growth factor (VEGF) or enhanced VEGF production by megakaryocytes (the platelet precursor cells). There has been an interest in non-haemostatic roles for platelets since the discovery that platelet depletion prevents growth of metastases [12]. Patients with cancers and inflammatory disorders that induce pathological angiogenesis demonstrate higher platelet levels of growth factors, including VEGF [13], [14]. It is known that the higher VEGF levels parallels tumour progression, and platelets are activated in the circulation of such individuals [15]. Lyst*^bg^* mice lacking dense granules and lysosomes demonstrate normal inflammatory angiogenesis [16], suggesting a role for α-granule release. Interestingly, pro-angiogenic and anti-angiogenic factors may be selectively released from platelets[17].

Evidence supporting the need for platelet adhesion includes unaffected *in vitro* endothelial tube formation in Matrigel by the addition of platelet secretate, which includes α-granule secretions, that is enhanced with the physical presence of platelets [18]. Also, platelet adhesion onto tumour endothelium is accompanied by VEGF release [19], and significantly more platelets have been observed adherent to angiogenic vessels compared to mature vasculature with intravital microscopy studies of dorsal skinfold chambers [20], supporting a role for platelet-vessel interactions. Whether platelets interact with endothelium or subendothelial structures remains unclear. While platelets bind subendothelial collagen after vessel injury during thrombosis, no evidence exists for a functional role of such interactions in physiological angiogenesis [21], nor for circulating platelet activation. Mice lacking GPVI, of central importance to direct platelet activation [22], demonstrate inflammatory angiogenesis in response to subcutaneous Matrigel implantation no differently from controls [16]. This suggests that intact collagen binding properties may not be required for angiogenesis to occur *in vivo*. Thus, platelet adhesion to endothelium may lead to angiogenesis as a result of releasate from activated platelets, but that secretion of pro-angiogenic factors may also occur without adhesion. For example, quiescent platelets may more effectively stimulate wound healing compared with activated platelets [23], possibly by enhancing fibroblast differentiation [24]. We therefore sought to identify a role for platelets using two *in vivo* models of physiological angiogenesis, sprouting and splitting forms of capillary growth [1] (Figure 1A), by examining the effect of platelet depletion by antibody treatment and pharmacological inhibition of platelet activation. We conclude that platelets differentially regulate mechanisms of angiogenesis *in vivo*.

## Materials and Methods

### *In vivo* experimentation

All procedures were performed in accordance with the UK Animals (Scientific Procedures) Act 1986, and approved by the University of Birmingham Biomedical Ethical Review Sub-committee. C57BL/6 (Charles River, Kent, UK), and MUC1-VEGF mice age and weight matched were used between 8–10 weeks of age (n = 4–9 per group). Weights did not differ between strains at 24.9±0.3 and 23.0±0.5 g respectively. Animals were housed at 21°C with 12:12 h light:dark cycle and *ad libitum* access to food and water. Longitudinal splitting angiogenesis was induced by 7 days oral *ad libitum* administration of 50 mg/L prazosin (Sigma, Poole, UK) a selective α_1_-adrenoceptor antagonist, in drinking water [3]. Induction of endothelial sprouting was induced by muscle overload [2]. Unilateral extirpation of alternate *m. tibialis anterior* between animals causes hyperplasia and hypertrophy in the remaining *m. extensor digitorum longus* (EDL) since the muscles are synergistic. Sampling occurred on the eighth day after surgery. Surgery was performed by initially anaesthetising mice with 5% isofluorane (Novartis Animal Health UK Ltd., Hertfordshire, UK) in O_2_ and sustaining anaesthesia with 2% isofluorane in O_2_. Wounds of approximately 1.5 cm were sutured using 6-0 absorbable suture. Systemic analgesic (2.5 mL/kg buprenorphine, s.c.; Temgesic NVS, National Veterinary Services Ltd., Stoke-on-Trent, UK) and topical antibiotic (Duplocillin LA, NVS) were administered perioperatively. See Figure 1B-C for protocol, and [40] for representative data in mice.

### Platelet depletion by antibody administration and pharmacological inhibition of platelet activation

Platelet depletion was achieved by single tail vein injection of 2 µg/g (4 µL/g) rat anti-mouse platelet glycoprotein Ibα (GPIbα) antibody (Emfret Analytics, Würzburg, Germany) or 4 µL/g 0.5% saline 24 h after overload or initiation of chronic vasodilatation [25], to permit coagulation of any bleeding during surgery. Antibody-induced platelet glycoprotein VI (GPVI) shedding was achieved by an identical regimen with rat anti-mouse GPVI antibody (Emfret) [26], dialysed to remove sodium azide preservative. Granulocytes/monocytes were depleted for 48 h with a single i.v. injection of 4 µg/g rat anti-mouse Ly6G/6C (Gr-1) antibody (BD Biosciences, Oxfordshire, UK) [27]. At 4, 24, and 48 h∼10 µl of whole blood was collected in a container containing acid citrate dextrose anti-coagulant following tail-bleed. Whole blood smears were generated and stained with Diff-Quik solution (Dade Behring, Newark, USA). Smears were visualised for presence of cells with lobed nuclei (neutrophils). Untreated animals undergoing granulocyte/monocyte depletion underwent exsanguination to generate smears.

Pharmacological inhibition of platelets was initially achieved by 30 mg/kg clopidogrel hydrogensulfate plus 30 mg/kg acetylsalicylic acid (aspirin, ASA; both from Sigma) [28] or either compound alone were dissolved in drinking water and administered 24 h after overload/prazosin initiation for 72 h (Figure SI in File S1). A lower dose of 15 mg/kg ASA (LD-ASA) was administered similarly. Adequate inhibition of cyclooxygenase activity was demonstrated in dose-sighting experiments (Figure SII in File S1).

### Tissue sampling for immunohistochemistry and VEGF protein quantification

Mice were killed by cervical dislocation. Muscles were immediately dissected and divided into two. One half was snap-frozen in liquid nitrogen for chemical analysis and the other in liquid nitrogen cooled isopentane (Sigma) mounted on 20 mm cork discs with OCT embedding matrix (both Raymond Lamb, Loughborough, UK) for sectioning. 10 µm sections for light microscopy were cut on a Bright Clinicut microtome at −20°C and mounted onto polysine™ slides (VWR International, Leuven, Belgium) and allowed to air dry. Samples were stored at −80°C and slides at −20°C.

For protein quantification, frozen tissue was powdered using a chilled pestle and mortar under liquid nitrogen, suspended in ice-cold lysis buffer (975 µl HEPES, 10 µl Protease Inhibitor Cocktail, 10 µl PMSF, 5 µl Na_3_Va_4_), and centrifuged at 5000 *g* for 20 min at 4°C. Samples were assayed in duplicate. Mouse and human VEGF protein (R&D Systems, Abingdon, UK), and PGF1α (Cambridge Biosciences, UK) was measured by enzyme linked immune sorbent assay (ELISA), according to manufacturer's instructions (5 animals/group). Total tissue protein concentration was determined by Bradford assay (using Bradford reagent, Sigma) performed in duplicate at the same time as the ELISA.

### Immunohistochemistry

**Figure 2:**
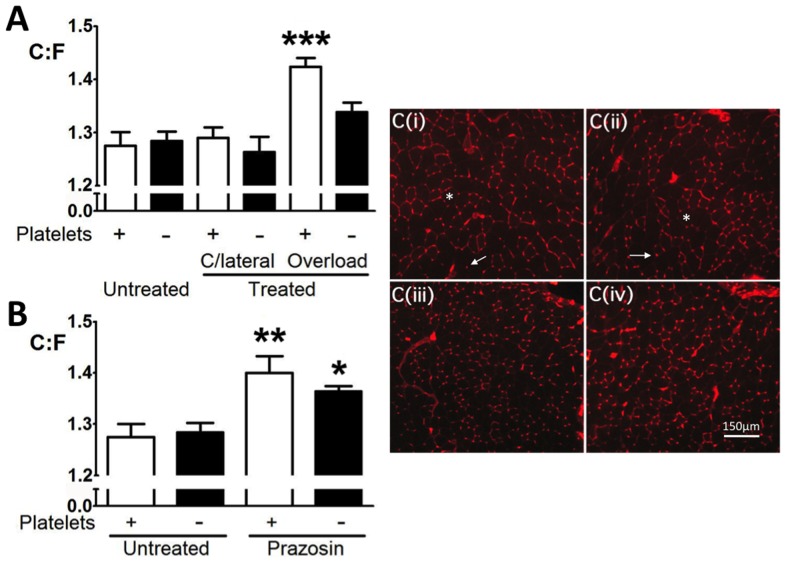
Platelet depletion differentially affects skeletal muscle angiogenesis. A) Capillary sprouting produced a significant capillary to fibre ratio (C∶F) increase, abolished by platelet depletion following i.p. injection of anti-GPIbα(denoted by ‘-’). Depletion did not affect C∶F of untreated or contralateral limbs. B) Longitudinal splitting caused a significant increase in C∶F which was unaffected by platelet depletion. *P<0.05, **P<0.01, ***P<0.001 *vs.* untreated. C) Detail of representative images of lectin-stained mouse muscle cross-section showing fibres (asterisks) and capillaries (arrows) for extirpation with (i) or without (ii) platelets, and prazosin with (iii) or without (iv) platelets.

Slides were warmed to room temperature (RT) for 30 min and staining performed on EDL sections fixed with 4% w/v electron microscopy grade formaldehyde (TAAB, Berkshire, UK). Following 3×5 min PBS washes capillary/sarcolemma staining used 1∶100 fluorescein *Griffonia simplicifolia* lectin-1 (Vector Laboratories Inc., Burlingame, CA): PBS for 1 h at RT in the dark was performed to permit quantification of capillary and fibre numbers (Figure 2C). Sections were washed in PBS and mounted on a coverslip with 1∶10 Vectorshield plus DAPI: PBS (Vector Laboratories Inc.). Sections were examined at ×10 magnification. Four fields of view per section were analysed. *In vivo* platelet labelling with DyLight488-labelled GPIbβ (Emfret Analytics, Würzburg, Germany) was performed by i.v. injection of 0.1 µg/g body weight 10 min prior to tissue sampling (performed as described above). Sections were examined at ×40 magnification. Imaging was performed using a Zeiss Axioskop 2 plus fluorescent microscope and images captured using an Axiocam MRc digital camera and Axiovision software (Karl Zeiss, UK). Capillary and fibre numbers were determined by counts after data blinding.

### BrdU pulse-labelling

The In Situ Cell Proliferation Kit, FLUOS (Roche, Mannheim, Germany) was used as per manufacturer's instructions. Briefly, animals were injected i.p. with bromo-deoxyuridine (BrdU) labelling reagent 16 h prior to tissue sampling. Immunostaining was also performed as instructed with the addition of 1∶100 rhodamine *Griffonia simplicifolia* lectin-1 (Vector Laboratories Inc., Burlingame, CA) to allow co-localisation of BrdU-labelled cells with vascular structures. Slides were mounted using 1∶10 Vectorshield plus DAPI: PBS to permit confirmation of nuclear localisation of BrdU-labelling.

### Generation of platelet-rich plasma and platelet isolation

To generate washed platelets, whole blood was taken slowly into a syringe contained 100 µl anticoagulant acid citrate dextrose. Blood was transferred to 1.5 ml tubes containing 200 µl 30°C Tyrodes solution (130 mM NaCl, 0.34 mM Na_2_HPO_4_, 2.9 mM KCl, 12 mM NaHCO_3_, 20 mM HEPES, 5 mM glucose, 1 mM MgCl_2_) at pH7.3. The blood was mixed and microcentrifuged at 500 *g* for 5 min, producing platelet rich plasma (PRP). PRP and around one third erythrocytes was removed to a new 1.5 ml tube and centrifuged at 120 *g* for 6 min before removal of PRP to a third 1.5 ml tube. To prevent aggregation 1 µl 10 mg/ml prostacyclin was added to PRP before centrifugation at 1000 *g* for 6 min. After supernatant removal, the platelet pellet was resuspended in 200 µl Tyrodes. Platelet numbers were determined with a Coulter counter (Beckman, High Wycombe, UK).

### Flow cytometry

Whole blood was taken into a syringe containing acid citrate dextrose anticoagulant from the inferior vena cava under 2% isofluorane anaesthesia, which does not affect platelet function [29], [30] to generate washed platelets before flow cytometry [31]. To determine GPVI expression following anti-GPVI administration 2 µl of FITC-conjugated antibody against GPVI (Emfret Analytics) was added to 100 µl of 1×10^6^ washed platelets resuspended in 30°C Tyrode's solution. Samples were rested for 30 min at RT in the dark. 200 µl PBS was added to stop the reaction, and samples analysed immediately.

### Statistical Analysis

Data are presented as Mean±SEM. Statistical analysis used one-way ANOVA with Bonferroni post hoc analysis, * = *P*<0.05, ** = *P*<0.01, and *** = *P*<0.001 *vs.* untreated control or contralateral muscle as appropriate.

## Results

### Platelet depletion impairs angiogenesis by capillary sprouting but not longitudinal splitting

Administration of the anti-GPIbα antibody led to a rapid loss of circulating platelets in mice. At 24 h post injection, circulating platelet numbers dropped to <5% of normal values, as we and others have previously reported [25], [31] (Table SI in File S1). Depletion was sustained for 48 h, with platelet numbers then rising to normal levels by 5 days post treatment (data not shown). Platelet depletion did not affect the capillary to fibre ratio (C∶F) of untreated animals, a robust means of quantifying angiogenesis since capillary numbers increase while fibre numbers remain consistent within a given area [32] (Figure SIII in File S1).

The first form of angiogenesis we examined was capillary sprouting. Surgical ablation of *m*. *tibialis anterior* causes overload of the synergist *m*. *extensor digitorum longus* (EDL), stimulating endothelial filopodia formation by mechanotransduction of lateral strain [1]. This represents angiogenesis under reduced fluid shear stress (FSS) relative to prazosin-induced longitudinal splitting. The C∶F increased by 11.7% at 7 days compared to contralateral muscles and muscles of untreated animals (*P*<0.001) (Figure 2A), consistent with our previous work [1]. Anti-GPIbα administration following induction of sprouting had no significant effect on the C∶F of contralateral muscles, but prevented the increase in the C∶F of ipsilateral muscles (n.s. *vs.* contralateral), indicating inhibition of sprouting angiogenesis.

**Figure 3:**
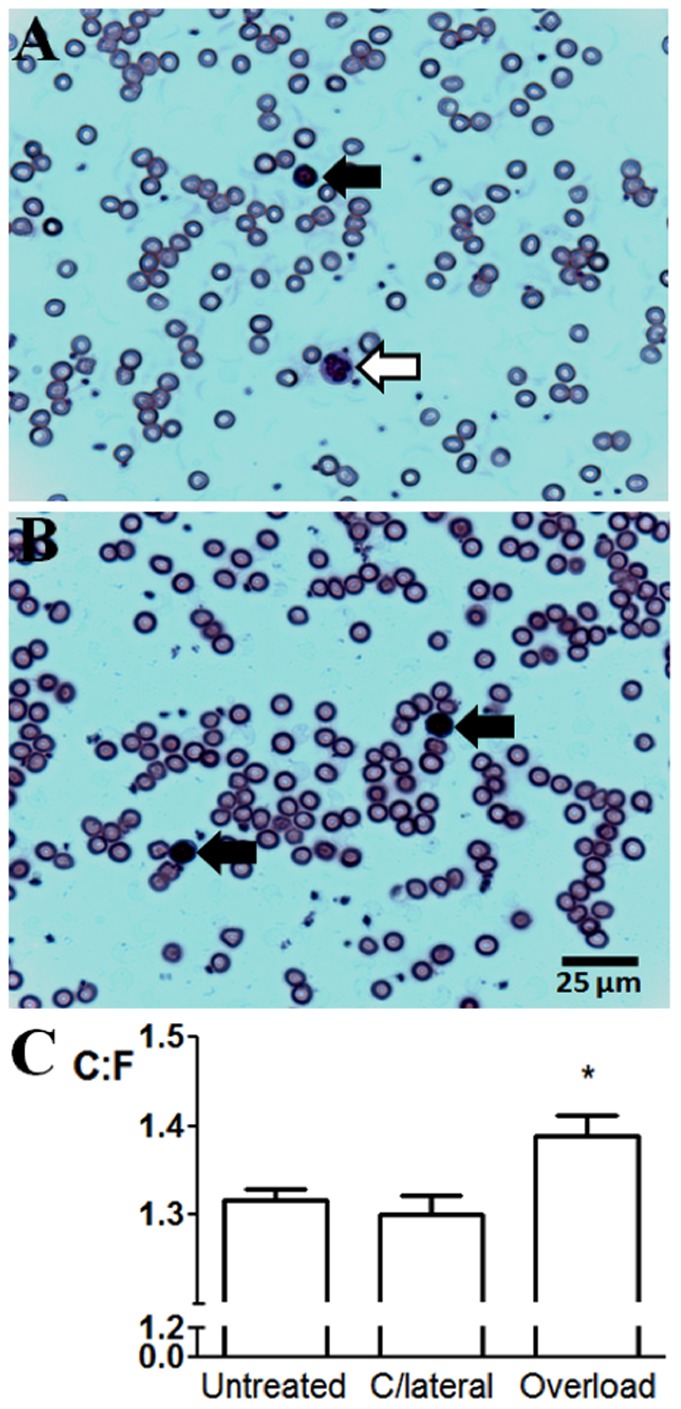
Granulocytes/monocytes do not mediate endothelial sprouting. A) Representative whole blood smears showing lymphocytes (black arrows) and granulocytes/monocytes (white arrow) were visualised in control animals. B) Following i.p. administration of anti-Ly6G/6C antibody only lymphocytes were readily visualised at 4, 24, and 48 h. C, Granulocyte/monocyte depletion did not alter the endothelial sprouting response normally observed (*P*<0.01).

Anti-GPIbα administration initiates an immune response by labelling platelets as foreign to the host, resulting in a rapid decrease in circulating leukocyte numbers during subsequent clearance of platelets, which had recovered by 24 h (Table SI in File S1). To confirm inhibition of sprouting angiogenesis resulted from *platelet* and not *leukocyte* depletion, we determined the effect of immune cell depletion alone. Administration of anti-Ly6G/6C causes depletion of granulocytes and monocytes lasting 48 h [27], but had no significant effect on platelet count (depleted 2.9×10^7^ *vs.* control 3.3×10^7^ platelets/ml total blood) or C∶F (Figure 3) of otherwise untreated animals. Further, immune cell depletion did not alter sprouting angiogenesis, showing a similar rise in C∶F as with untreated mice (*P*<0.05). These data indicate that the reduction in immune cell numbers resulting from platelet depletion did not contribute to the inhibition of angiogenesis.

Angiogenesis induced by chronic vasodilatation leads to capillary growth through mechanotransduction of elevated FSS [3]. Induction of longitudinal splitting led to a 9.8% higher C∶F in EDL after 7 days (*P*<0.01), in line with our previous work [1], with C∶F also raised following platelet depletion (*P*<0.05; Figure 2B), suggesting the mechanism of longitudinal splitting angiogenesis is platelet-independent.

### Activation of platelets *via* COX1 but not P2Y_12_ is required to promote capillary sprouting angiogenesis

**Figure 4:**
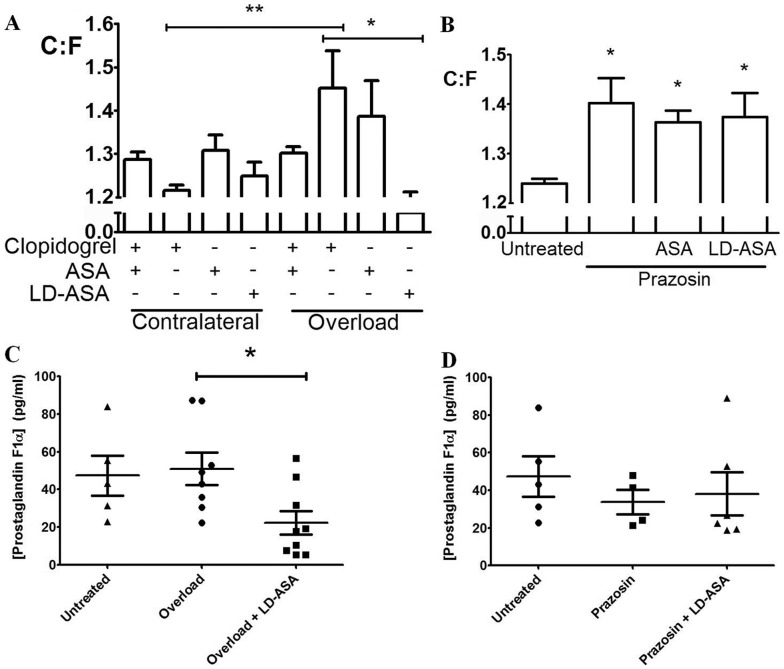
Platelets differentially affect angiogenesis *via* COX signalling. A) Dual clopidogrel/ASA regimen inhibited sprouting angiogenesis. Single regimens identified ASA as the active agent, with clopidogrel unable to alter the angiogenic response. B) Longitudinal splitting was unaffected by ASA or lower dose (LD)-ASA. C) PGF1α dropped significantly with overload + LD-ASA. D) Induction of longitudinal splitting did not alter PGF1α levels. *P<0.05, **P<0.01 between columns (A), or *vs.* untreated controls (B).

Having demonstrated that the induction of sprouting angiogenesis likely involves platelet mediation, we explored possible mechanisms by pharmacological inhibition of platelet activation. A combined regimen of clopidogrel hydrogensulfate with acetylsalicylic acid (ASA; aspirin) at varying concentrations is used clinically to inhibit platelet activation in order to limit thrombosis [33]. Clopidogrel antagonises the platelet specific P2Y_12_ receptor. ASA irreversibly inhibits both cyclooxygenase (COX) 1 and 2, preventing thromboxane A2 (TXA_2_) and prostacyclin (PGI_2_) production, although platelets express COX1 alone. Dual clopidogrel/ASA treatment at doses of 30 mg/L for each drug blocked capillary sprouting angiogenesis (Figure 4A), supporting our findings with anti-GPIbα administration.

To clarify the underlying mechanism we induced sprouting angiogenesis with either clopidogrel or 30 mg/L ASA treatment alone. Clopidogrel treatment led to capillary sprouting (19.8% increase in C∶F, *P*<0.01) similar to animals subjected to muscle overload without drug administration, suggests P2Y_12_ signalling is not involved in sprouting angiogenesis. However, ASA markedly suppressed the angiogenic response to overload (Figure 4A), suggesting inhibition of TXA_2_ or PGI_2_ was responsible for the dual regimen inhibition of capillary sprouting.

A lower ASA dose (LD-ASA) was also investigated, as this is likely to generate a more platelet-specific response, which showed similar results to the higher dose ASA (Figure 4A). LD-ASA inhibited sprouting angiogenesis (*P*<0.05), with no significant difference in the C∶F observed (*P*<0.05). Quantification of the PGI_2_ breakdown product PGF1α in plasma demonstrated a significant decrease in levels at day 7 (*P*<0.05) following LD-ASA treatment with overload (Figure 4C). We are therefore unable to distinguish between possible mediation of sprouting angiogenesis by TXA_2_ or PGI_2_, even at lower concentrations of ASA. In contrast to the anti-angiogenic effects, inhibition of platelet activation with ASA is dose-dependent (Figure SII in File S1).

We also inhibited COX-mediated platelet activation by ASA and LD-ASA from 24–96 h after initiation of longitudinal splitting, which demonstrated no effect with platelet depletion. ASA and LD-ASA treatment had no effect on the normal difference seen in the C∶F ratio (both *P*<0.05; Figure 4B), paralleling the anti-GPIbα finding and adding further support to the hypothesis that platelets do not mediate longitudinal splitting. Induction of longitudinal splitting had no effect on plasma levels of PGF1α (Figure 4D), suggesting the chronic increase in FSS sustained PGI_2_ levels but did not enhance them.

### Increased muscle VEGF does not compensate for platelet depletion in sprouting angiogenesis

Previous reports suggest release of growth factors from secreted α-granules mediate pathological forms of sprouting angiogenesis [34]. VEGF is considered a key mediator of both pathological and physiological angiogenesis, and is contained within platelet α-granules [15]. Thus, inhibition of capillary sprouting by platelet depletion or inhibition could result from the absence or impairment of local VEGF delivery. To test this hypothesis, we determined whether tissue overexpression of VEGF compensated for loss of platelet VEGF after platelet depletion. We used a transgenic mouse line, MUC1-VEGF, expressing human VEGF_121_ in addition to murine VEGF isoforms, resulting in significantly higher VEGF levels in all tissues examined [35]. Although contribution to tissue levels of VEGF by resident platelets is likely extremely low in the absence of adhesion, we determined platelet [VEGF]. Levels of the murine isoform were similar among strains, while human isoform expression was below ELISA detection limits (Table SII in File S1).

**Figure 5:**
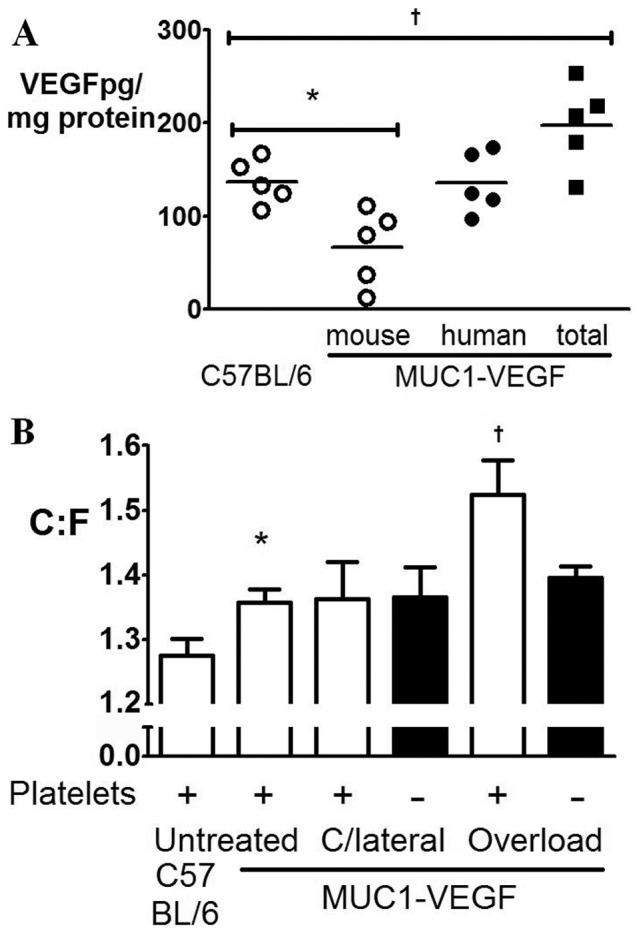
VEGF overexpression cannot rescue angiogenic deficit. A) Extensor digitorum longus muscle VEGF expression in wildtype and MUC1-VEGF mice. MUC1-VEGF human VEGF (•) plus mouse VEGF (○) resulted in a 45% increase in total content (▪) *vs.* wildtype. B) MUC1-VEGF mice had higher initial C∶F than wildtype. Capillary sprouting with or without platelets was no different from wildtype. *P<0.05 *vs.* C57 BL/6 controls, +*P*<0.05 *vs.* contralateral.

Compared to wildtype, MUC1-VEGF EDL muscles demonstrated ∼50% higher overall (mouse + human) VEGF expression (Figure 5A). Untreated MUC1-VEGF mice had a higher C∶F than wildtype animals (*P*<0.05), presumably resulting from chronic exposure to elevated VEGF. Following induction of sprouting angiogenesis, MUC1-VEGF mice increased the C∶F ratio (*P*<0.05) compared to contralateral muscles to similar levels observed in wildtype animals (11.8% *vs.* 11.7% in wildtype mice). Further, platelet depletion by anti-GPIbα administration led to an inhibition of capillary sprouting comparable to that occurring in wildtype animals (Figure 5B). VEGF_121_ overexpression is therefore unable to rescue sprouting angiogenesis following platelet depletion, indicating a more substantive role for platelets than α-granule secretion and local VEGF delivery.

### Platelet mediation of capillary sprouting is independent of collagen-induced platelet activation

**Figure 6:**
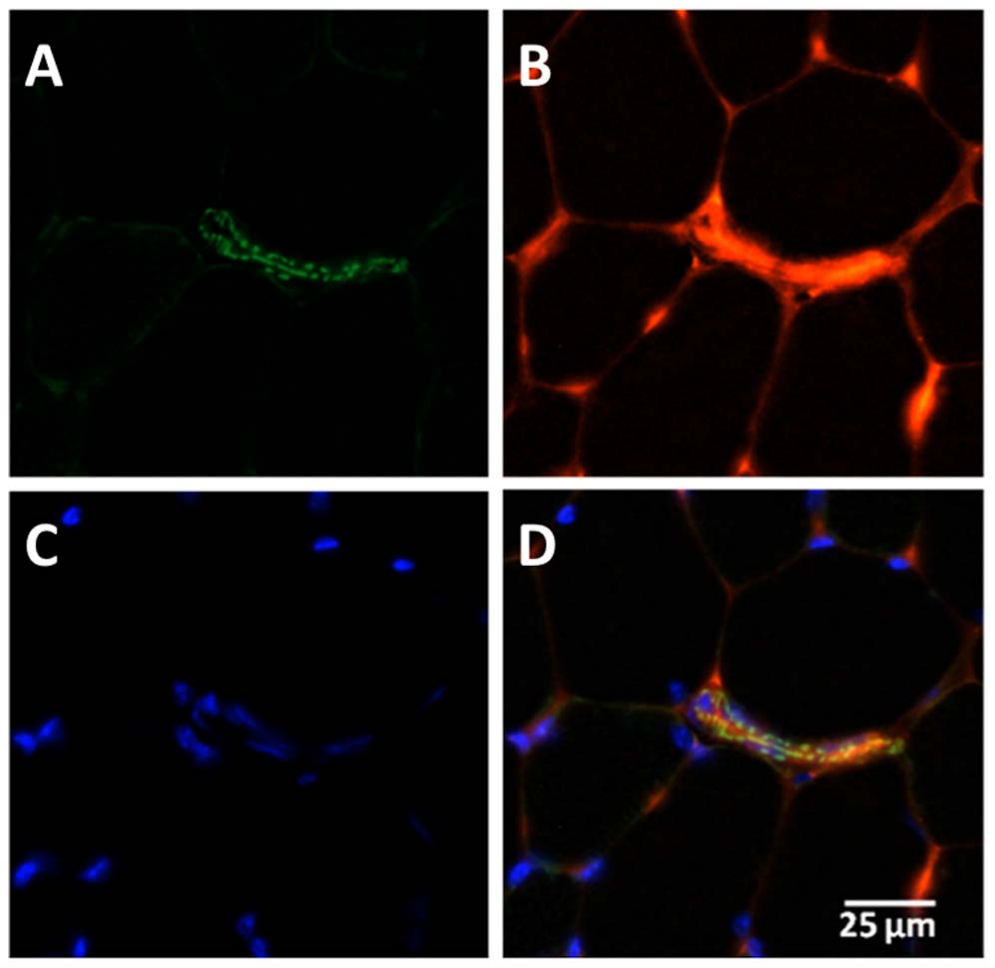
Platelets are observable in skeletal muscle vasculature. Platelets were labelled *in vivo* prior to muscle sampling. A) Imaging of muscle sections for the presence of platelets occurred under fluorescent microscopy in conjunction with B) rhodamine *Griffonia simplicifolia* lectin-1 staining for visualisation of vasculature, and C) DAPI for localisation of nuclei. D) A merged image of the three other panels depicts platelets within a venule, a potential locus for angiogenesis.

**Figure 7:**
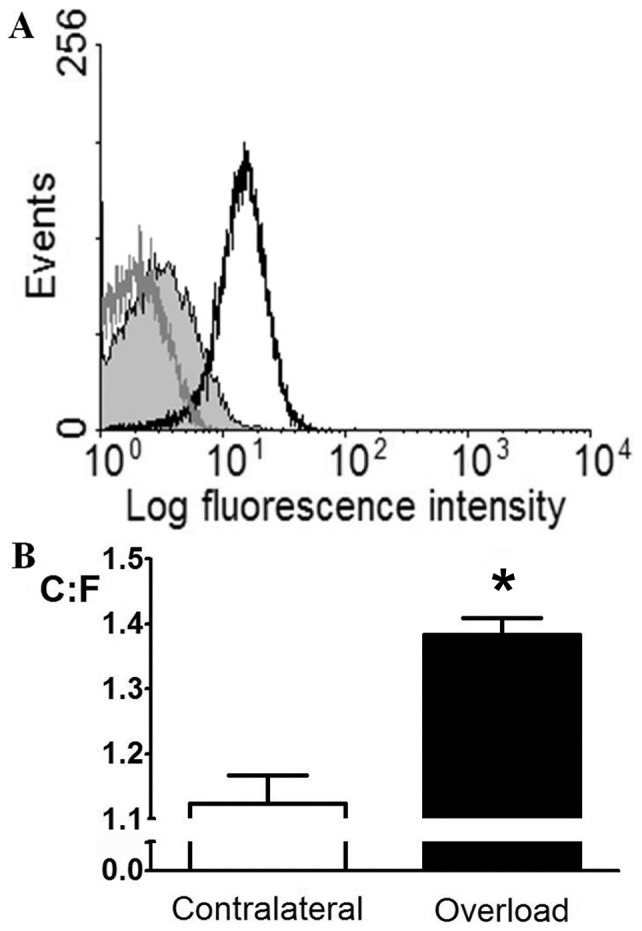
Angiogenesis is independent of collagen-induced platelet activation. A) Anti-GPVI resulted in ∼70% GPVI shedding. Shaded grey peak, GPVI shed; clear black peak, control; clear grey peak, GPVI shed IgG expression. B) GPVI shedding did not alter capillary sprouting, with significantly increased C∶F. *P<0.05 *vs.* contralateral.

Since compensation for loss of platelet VEGF did not rescue sprouting angiogenesis we investigated whether platelet mediation required platelet-collagen interactions, as would occur during thrombosis, although no evidence has yet been found for collagen exposure during capillary sprouting [21]. *In vivo* platelet labelling at early (2 day) and later (7 day) stages of capillary growth confirmed that platelets can be observed within the vasculature (Figure 6), but were not localised to any specific element of the neovasculature. Indeed, we could identify no difference in platelet content of angiogenic loci between untreated and treated animals (data not shown). However, to exclude the requirement for activation following platelet-collagen interactions we induced sprouting angiogenesis followed by administration of an anti-GPVI antibody. The antibody caused shedding of about 70% GPVI as assessed by flow cytometry analysis at 7 days (Figure 7A). GPVI is of central importance to direct platelet activation by collagen, irrespective of FSS level [22], but shedding did not alter the extent of sprouting angiogenesis (C∶F *P*<0.05 *vs.* contralateral control, Figure 7B). That loss of GPVI had no effect on capillary sprouting is consistent with the lack of an effect on pathological angiogenesis in GPVI knockout mice [16].

### Platelet depletion has no effect on proliferation of capillary-associated cells in capillary sprouting

**Figure 8:**
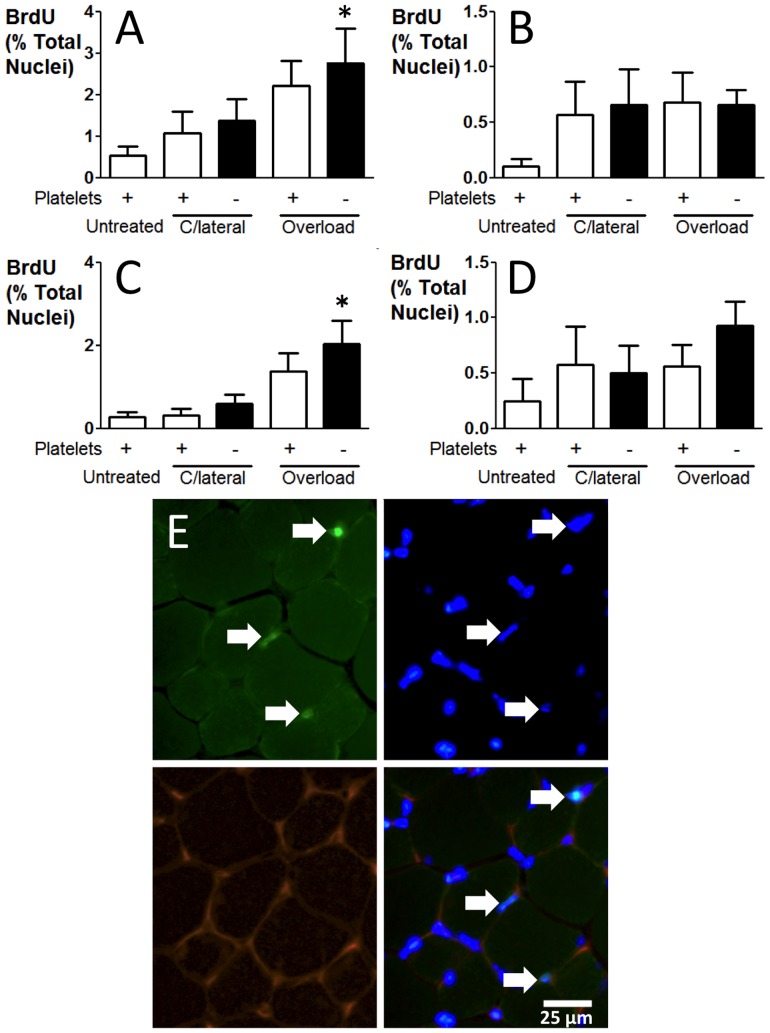
Platelet depletion did not affect proliferation of capillary-associated cells or myocytes. A) The total number of proliferating cells increased slightly in tissue after induction of capillary sprouting, but was not affected by platelet depletion. B) Capillary-associated cell proliferation was unaffected by platelet depletion. C) The increase in proliferating cell number with sprouting results from increased interstitial cell proliferation. D) The absence of platelets did not impair the proliferation of myocytes following muscle overload. E) BrdU labelling was performed in conjunction with rhodamine *Griffonia simplicifolia* lectin-1 staining (bottom left panel) for visualisation of vasculature, and DAPI (top right panel) to ensure BrdU staining observed was localised to nuclei. A merge (bottom right panel) of the three other panels with three BrdU-labelled interstitial cells (white arrows) is shown. Imaging was performed with fluorescent light microscopy as described in the methods at ×40 magnification. *P<0.05 *vs.* contralateral.

The total number of BrdU positive cells showed little increase following induction of muscle overload, although this reached statistical significance when induction of capillary sprouting followed platelet depletion (*P*<0.05) this still represented only ∼3% of the total nuclei content (Figure 8A). We divided total BrdU counts into capillary-associated (endothelium, mural cells; Figure 8B), interstitial cells (e.g. fibroblasts; Figure 8C), or myocytes (Figure 8D) by co-localisation with rhodamine-linked isolectin B4. The increase in BrdU labelling after capillary sprouting resulted primarily from increased proliferation of interstitial cells at 7 days (Figure 8E). Numbers of proliferative capillary-associated cells were similar in sprouting angiogenesis with or without platelet depletion (Figure 8B). These data demonstrate that platelet depletion is not anti-angiogenic due to impaired EC proliferation, suggesting platelets may promote capillary sprouting *via* inhibition of another component of angiogenesis such as matrix proteolysis or EC migration.

## Discussion

Platelets are normally regarded as effectors of haemostasis, but proteomic analyses suggest they may play a wider role in wound healing, tumour growth, inflammation and regeneration [36]. Our data show a differential role for platelets in mediating two distinct mechanisms of *in vivo* angiogenesis, namely capillary sprouting and longitudinal splitting. A failure to rescue platelet depletion by VEGF overexpression suggests the mechanism is more complex than α-granule secretion alone. It is difficult to directly assess platelet granule release *in vivo*, and monocyte-derived VEGF may play a role in some forms of angiogenesis [37], but these data are consistent with VEGF levels *per se* (irrespective of the source) not being responsible for angiogenesis ablation following platelet depletion. Maintenance of angiogenesis following loss of GPVI expression, although partial, indicates collagen-induced platelet activation is not the primary stimulus, while BrdU pulse-labelling suggests platelets do not affect endothelial cell (EC) proliferation.

Sprouting angiogenesis induced by muscle overload [2] causing elevated mechanical deformation of vessels and a resulting reduction in capillary FSS [1], was abolished by platelet depletion. In contrast, splitting angiogenesis is induced by elevated capillary FSS [3], and was unaffected by platelet depletion. Together, these data may suggest a shear-related response to platelet mediation of physiological angiogenesis. It is well known that higher FSS levels induce upregulation of vasoprotective genes in EC [38]. Indeed, while we observed significantly decreased levels of PGF1α with capillary sprouting there was no alteration in levels with longitudinal splitting. An alternative hypothesis is a difference in signalling between the angiogenic forms. For example, sprouting is dependent on matrix metalloprotease activity [39] while both forms require VEGF [40]. VEGF is found in both platelets and granulocytes, particularly neutrophils [41]. However, capillary sprouting was normal following granulocyte/monocyte depletion, demonstrating that despite the initial fall in immune cell numbers during platelet clearance after anti-GPIbα administration, it was depletion of platelets rather than immune cells that mediated angiogenesis.

Inhibition of capillary sprouting following treatment with a dual regimen of clopidogrel/ASA confirmed the platelet depletion result. ASA was identified as the active agent at both high and low dose regimens. The data are consistent with overload-induced angiogenesis acting through COX signalling, and implicate COX inhibition of TXA_2_ or PGI_2_ synthesis as the anti-angiogenic target. Although ASA is not platelet specific, treatment may have a greater effect on platelets than EC depending on dose treatment regimen, since anucleate platelets are unable to initiate COX gene transcription. In addition, EC are primarily responsible for production of PGI_2_, and platelets TXA_2_, so that while PGI_2_ levels are able to recover in this model, inhibition of TXA_2_ is irreversible [42], suggesting that TXA_2_ may be the target molecule. Our data suggests a graded response to different ASA doses with LD-ASA resulting in C∶F similar to untreated animals, while ASA doses showed an increase. Further studies are required to fully define the *in* vivo mechanisms involved. Splitting angiogenesis was unaffected by ASA and LD-ASA treatment, confirming this form of angiogenesis does not require platelet mediation.

Having identified a role for platelets in physiologically-induced sprouting angiogenesis we sought to identify whether mediation required α-granule secretion alone or platelet-collagen interactions as with thrombosis. Animals lacking either platelet dense granules or lysosomes undergo angiogenesis in a manner unchanged from controls [16], suggesting α-granules are required for mediation of capillary sprouting. Studies into platelet mediation of pathological angiogenesis has concentrated on the role of VEGF, contained within α-granules [15] and widely seen as the most important growth factor to capillary growth. Indeed, endothelial sprouting is abolished without VEGF [40].

We reasoned that if α-granule content was responsible for mediation of capillary sprouting, compensating for the loss of platelet-derived VEGF after depletion may rescue the angiogenic response. We therefore induced sprouting angiogenesis in MUC1-VEGF transgenic mice, which have tissue VEGF levels 45% greater than wildtype mice due to expression of human VEGF_121_ in addition to normal mouse isoforms. Although this overexpression was not detectable in platelets, levels of murine VEGF were similar to wildtype and hence we could detect any influence of altered tissue concentrations. In contrast to studies showing reliance of angiogenesis in VEGF, compensation of VEGF levels by overexpression did not rescue capillary sprouting, which was abolished in both wildtype and MUC1-VEGF mice with platelet depletion. Subsequent to submission of the original manuscript, intriguing *in vitro* data has suggested that platelet mediated angiogenesis is inhibited by ASA and may be independent of VEGF [43].

Capillary sprouting is therefore unlikely to be driven by release of VEGF from platelet α-granules, and so we tested the hypothesis that platelet-vessel interactions are responsible. During thrombosis, platelets become activated by exposed subendothelial collagen through GPVI. Mice with approximately 70% of GPVI shed demonstrated capillary sprouting in a similar manner to wildtype mice, suggesting collagen binding is not a significant mechanistic pathway during this form of angiogenesis. Indeed, we have previously identified no subendothelial exposure by electron micrograph analysis [21]. Our GPVI data is consistent with data from GPVI knockout mice implanted with subcutaneous Matrigel to model inflammatory angiogenesis, [16], where no change was observed.

Our data suggests a requirement for platelets at the initiation phase of the angiogenic response, since platelet depletion early on inhibits angiogenesis even 5 days after platelet numbers have normalised [25], [31]. The increase in number of proliferating cells following induction of capillary sprouting remained after platelet depletion. Co-localisation analysis demonstrated that the labelling index of capillary-associated cells and myocytes remained unchanged after capillary sprouting with or without platelet depletion. The increase in BrdU-labelling was therefore a result of increased proliferation of interstitial cells, and indicates that platelet depletion is not anti-angiogenic due to inhibition of EC proliferation. It is presumed, therefore, that platelets promote capillary sprouting *via* activation of another component of angiogenesis, such as endothelial migration and/or proteolytic modification of the extracellular matrix. In fact, inhibition of matrix metalloproteases required for extracellular matrix breakdown abolishes sprouting angiogenesis [39]. Additionally, it is conceivable that sphingosine-1-phosphate is required, since TXA_2_ synthesis and thromboxane receptor activation mediate its release, while ASA inhibits its release [44].

In conclusion, using two distinct forms of capillary growth *in vivo* we have identified differential platelet mediation of physiological angiogenesis. Angiogenesis in high FSS environments is platelet independent, whereas angiogenesis under lower FSS is platelet mediated. Endothelial sprouting requires the presence of platelets, but not in delivery of pro-angiogenic growth factors released from α-granules alone, or platelet interactions with collagen. Platelet mediation did not result from alterations in proliferative potential of endothelium or perivascular cells, but platelet activation *via* COX is fundamental to sprouting angiogenesis.

## Supporting Information

File S1 **Table SI, Differential white cell count 24 h following platelet depletion (×10^3^.mm^−3^). Table SII, Platelets VEGF content is not altered by treatment (pg VEGF.10^−6^ platelets; mean±SEM).** **Figure SI, Experimental protocol for inhibition of platelet activation with clopidogrel and/or acetylsalicylic acid (ASA; aspirin).** A) Endothelial sprouting was induced by extirpation of *m*. *tibialis anterior* causing overload of *m*. *extensor digitorum longus*. 24 h after surgery oral 30 mg/kg clopidogrel/ASA as dual or single regimens was administered for 72 h. B) ASA administration was begun 24 h after initiation of prazosin treatment, lasting 72 h. Prazosin induces longitudinal splitting. In both A and B, sampling occurred on the eighth day. **Figure SII, Inhibition of platelet activation with acetylsalicylic acid (ASA; aspirin) is dose-dependent.** Mice were administered aspirin *via* drinking water for 7 days. Experimental groups were 3 control mice, 3 low dose aspirin mice (30 mg/litre) and 3 high dose aspirin mice (300 mg/litre). Following 7 days aspirin treatment, blood was collected into sodium citrate and platelet-rich plasma (PRP) was prepared by centrifugation. Platelet aggregation was monitored by light transmission aggregometry in a Born lumi-aggregometer. Following stimulation with 0.5 mM arachidonic acid, platelets from the low dose aspirin treated mice aggregated normally (A) whereas platelets from the high dose aspirin treated mice showed only a minimal shape change response (B). Platelets from all mice aggregated normally to 500 mM PAR-4 peptide (C). **Figure SIII, Influence of scaling on muscle capillary supply.** In response to reviewer comments we offer an explanation for the choice of capillary to fibre ratio (C∶F) as the most robust index of angiogenesis. It has been repeatedly been shown that other methods of quantifying angiogenesis (such as capillary density; Hudlická O, Brown MD, Egginton S. 1992. Angiogenesis in skeletal and cardiac muscle. Physiological Reviews 72: 369–417) have a bias due to alterations in fibre size often observed among experimental groups, that mask the changes in capillary number, whereas C∶F is much less sensitive to these scaling effects (Egginton S. 1990. Morphometric analysis of tissue capillary supply. In: Boutilier RG (ed) Vertebrate Gas Exchange from Environment to Cell. Advances in Comparative and Environmental Physiology. 6: 73–141; Hudlická O, Brown MD, Egginton S. 1998. Angiogenesis: basic concepts and methodology. Chapt. 1, pp 3–19. In: Halliday A, Hunt BJ, Poston L, Schachter M (eds) An Introduction to Vascular Biology. CUP). The current experiment is no different: (1) contralateral muscle ± platelet depletion show minor, but reciprocal differences in CD and a(f). (2) extirpation without platelet depletion has a similar CD to contralateral muscle despite muscle hypertrophy that would be expected to reduce CD if angiogenesis had not occurred (C∶F is significantly higher); in contrast extirpation with platelet depletion had both a similar CD and a(f) as contralateral muscles reflecting an absence of angiogenesis (C∶F similar). (3) in the same way, relatively small differences in fibre size explain the modest difference in CD among prazosin groups, while the C∶F is very similar (see Fig. 2 in the main text). Abbreviations: CD, capillary density; a(f), fibre cross-sectional area; C-, contralateral without platelet depletion; C+, contralateral with platelet depletion; E, extirpation; P, prazosin.
(DOC)
