# Supplementary material for: In Vivo Evidence for Platelet-Induced Physiological Angiogenesis by a COX Driven Mechanism
Source: PLoS One. 2014 Sep 19;9(9):e107503. doi: 10.1371/journal.pone.0107503 (PMC4169573; doi:10.1371/journal.pone.0107503)
Supplement: File S1 — Table SI, Differential white cell count 24 h following platelet depletion (×103.mm−3). Table SII, Platelets VEGF content is not altered by treatment (pg VEGF.10−6 platelets; mean±SEM). Figure SI, Experimental protocol for inhibition of platelet activation with clopidogrel and/or acetylsalicylic acid (ASA; aspirin). A) Endothelial sprouting was induced by extirpation of m. tibialis anterior causing overload of m. extensor digitorum longus. 24 h after surgery oral 30 mg/kg clopidogrel/ASA as dual or single regimens was administered for 72 h. B) ASA administration was begun 24 h after initiation of prazosin treatment, lasting 72 h. Prazosin induces longitudinal splitting. In both A and B, sampling occurred on the eighth day. Figure SII, Inhibition of platelet activation with acetylsalicylic acid (ASA; aspirin) is dose-dependent. Mice were administered aspirin via drinking water for 7 days. Experimental groups were 3 control mice, 3 low dose aspirin mice (30 mg/litre) and 3 high dose aspirin mice (300 mg/litre). Following 7 days aspirin treatment, blood was collected into sodium citrate and platelet-rich plasma (PRP) was prepared by centrifugation. Platelet aggregation was monitored by light transmission aggregometry in a Born lumi-aggregometer. Following stimulation with 0.5 mM arachidonic acid, platelets from the low dose aspirin treated mice aggregated normally (A) whereas platelets from the high dose aspirin treated mice showed only a minimal shape change response (B). Platelets from all mice aggregated normally to 500 mM PAR-4 peptide (C). Figure SIII, Influence of scaling on muscle capillary supply. In response to reviewer comments we offer an explanation for the choice of capillary to fibre ratio (C∶F) as the most robust index of angiogenesis. It has been repeatedly been shown that other methods of quantifying angiogenesis (such as capillary density; Hudlická O, Brown MD, Egginton S. 1992. Angiogenesis in skeletal and cardiac muscle. Physiological Reviews 72: 369 [file pone.0107503.s001.doc]

**Table SI.** **Differential white cell count 24 h** **following platelet depletion (x10^3.^mm^-3^).**

Control	Depleted

Platelets	888.40±21.01	18.28±0.001**

Lymphocytes	2.42±0.07	2.81±0.002*

Monocytes	0.04±0.01	0.09±0.001**

Neutrophils	0.15±0.04	0.13±0.06

Eosinophils	N/D	N/D

Basophils	0.01±0.01	0.01±0.001

Total WBC	2.61±0.07	3.08±0.001**

* *P*<0.01, ***P*<0.001 *vs.* control

**Table SII. Platelets VEGF content is not altered by treatment (pg VEGF^.^10^-6^ platelets; mean±SEM).**

mVEGF	hVEGF

Wildtype (n=5)	0.57±0.08	N/D

MUC1 (n=7)	0.68±0.15	N/D

Murine blood was drawn by cardiac puncture from mice terminally narcosed with CO_2_ and taken into 100µl acid citrate dextrose (ACD). Platelet rich plasma (PRP) and the top third of the erythrocyte layer was obtained by centrifugation at <200g for 5 minutes at room temperature. PRP was isolated by further centrifugation at 200g to remove remaining erythrocytes. Platelets were pelleted at 1000g for 6 minutes and resuspended in modified Tyrode’s buffer (5mM HEPES, 136mM NaCl, 2.7mM KCl, 0.42mM NaH_2_PO_4_.H_2_O, 2mM MgCl_2_, 5.6mM glucose, 0.1% BSA) to a cell density of 1-5 x10^8^/ml. Platelets were counted by Beckman Coulter counter (Beckman Coulter, Particle Analyser Z2). Washed platelets were lysed in 2x platelet lysis buffer (2% NP40, 30 mM Hepes, 150 mM NaCl, 2 mM EDTA, pH 7.4) for 30 minutes at 4ºC. Cell debris and DNA were removed by centrifugation. Human VEGF was measured using the Human VEGF Quantikine ELISA kit (R&D systems, DVE00) and murine VEGF was measured using the Mouse VEGF Quantikine ELISA kit (R&D systems, MMV00) according to the manufacturer’s instructions. VEGF levels were corrected for background signal and compared to standard concentrations of VEGF; all values are expressed as pg VEGF per 1x10^6^ platelets assayed.

**Figure SI. Experimental protocol for inhibition of platelet activation with clopidogrel and/or acetylsalicylic acid (ASA; aspirin).** A) Endothelial sprouting was induced by extirpation of *m. tibialis anterior* causing overload of *m. extensor digitorum longus*. 24 h after surgery oral 30 mg/kg clopidogrel/ASA as dual or single regimens was administered for 72 h. B) ASA administration was begun 24 h after initiation of prazosin treatment, lasting 72 h. Prazosin induces longitudinal splitting. In both A and B, sampling occurred on the eighth day.

**Figure SII. Inhibition of platelet activation with acetylsalicylic acid (ASA; aspirin) is dose-dependent.** Mice were administered aspirin *via* drinking water for 7 days. Experimental groups were 3 control mice, 3 low dose aspirin mice (30 mg/litre) and 3 high dose aspirin mice (300 mg/litre). Following 7 days aspirin treatment, blood was collected into sodium citrate and platelet-rich plasma (PRP) was prepared by centrifugation. Platelet aggregation was monitored by light transmission aggregometry in a Born lumi-aggregometer. Following stimulation with 0.5 mM arachidonic acid, platelets from the low dose aspirin treated mice aggregated normally (A) whereas platelets from the high dose aspirin treated mice showed only a minimal shape change response (B). Platelets from all mice aggregated normally to 500 mM PAR-4 peptide (C).

**Figure SIII. Influence of scaling on muscle capillary supply.** In response to reviewer comments we offer an explanation for the choice of capillary to fibre ratio (C:F) as the most robust index of angiogenesis. It has been repeatedly been shown that other methods of quantifying angiogenesis (such as capillary density; Hudlická O, Brown MD, Egginton S. 1992. Angiogenesis in skeletal and cardiac muscle. Physiological Reviews 72:369-417) have a bias due to alterations in fibre size often observed among experimental groups, that mask the changes in capillary number, whereas C:F is much less sensitive to these scaling effects (Egginton S. 1990. Morphometric analysis of tissue capillary supply. In: Boutilier RG (ed) Vertebrate Gas Exchange from Environment to Cell. Advances in Comparative and Environmental Physiology. 6:73-141; Hudlická O, Brown MD, Egginton S. 1998. Angiogenesis: basic concepts and methodology. Chapt. 1, pp 3-19. In: Halliday A, Hunt BJ, Poston L, Schachter M (eds) An Introduction to Vascular Biology. CUP). The current experiment is no different: (1) contralateral muscle ± platelet depletion show minor, but reciprocal differences in CD and a(f). (2) extirpation without platelet depletion has a similar CD to contralateral muscle despite muscle hypertrophy that would be expected to reduce CD if angiogenesis had not occurred (C:F is significantly higher); in contrast extirpation with platelet depletion had both a similar CD and a(f) as contralateral muscles reflecting an absence of angiogenesis (C:F similar). (3) in the same way, relatively small differences in fibre size explain the modest difference in CD among prazosin groups, while the C:F is very similar (see Fig. 2 in the main text). Abbreviations: CD, capillary density; a(f), fibre cross-sectional area; C-, contralateral without platelet depletion; C+, contralateral with platelet depletion; E, extirpation; P, prazosin.
